# The Long-Term Effect of Weight Loss on the Prevention of Progression to Cirrhosis among Patients with Obesity and MASH-Related F3 Liver Fibrosis

**DOI:** 10.3390/ijerph21060708

**Published:** 2024-05-30

**Authors:** Jiafei Niu, Wael Al-Yaman, Kanokwan Pinyopornpanish, Ji Seok Park, Miguel Salazar, Huijun Xiao, James Bena, Ruishen Lyu, Gianina Flocco, Shilpa R. Junna, Talal Adhami, Omar T. Sims, Jamile Wakim-Fleming

**Affiliations:** 1 Department of Gastroenterology, Hepatology and Nutrition, Cleveland Clinic Foundation, Cleveland, OH 44106, USA; niuj@ccf.org (J.N.); walyaman@gmail.com (W.A.-Y.); kpinyopornpanish@gmail.com (K.P.); parkj6@ccf.org (J.S.P.); miguel.j.salazar88@gmail.com (M.S.); floccog2@ccf.org (G.F.); junnas@ccf.org (S.R.J.); adhamit@ccf.org (T.A.); fleminj1@ccf.org (J.W.-F.); 2 Department of Gastroenterology, St. Joseph Mercy Ann Arbor Hospital, Ypsiilanti, MI 48197, USA; 3 Division of Gastroenterology, Department of Internal Medicine, Faculty of Medicine, Chiang Mai University, Chiang Mai 50200, Thailand; 4 Department of Quantitative Health Sciences, Lerner Research Institute, Cleveland Clinic Foundation, Cleveland, OH 44106, USA; xiaoh@ccf.org (H.X.); benaj@ccf.org (J.B.); ruishenlyu52@gmail.com (R.L.)

**Keywords:** obesity, weight loss, fibrosis, MASH-related fibrosis, cirrhosis

## Abstract

This multi-center retrospective study examined the effect of weight loss on the prevention of progression to cirrhosis in a sample exclusively composed of patients with obesity and MASH-related F3 liver fibrosis. Adult patients with obesity and biopsy-confirmed MASH-related F3 liver fibrosis (*n* = 101) from two liver transplant centers in the US were included in the study. A higher proportion of patients who did not progress to cirrhosis achieved >5% weight loss at follow-up (59% vs. 30%, *p* = 0.045). In multivariable analysis, patients with >5% weight loss at follow-up had a lower hazard of developing cirrhosis compared to patients with no weight loss or weight gain (HR: 0.29, 95%, CI: 0.08–0.96); whereas, diabetes (HR: 3.24, 95%, CI: 1.21–8.67) and higher LDL levels (HR: 1.02, 95%, CI: 1.01–1.04) were associated with higher hazards of progression to cirrhosis. Weight loss >5% has the potential to prevent disease progression to cirrhosis in patients with obesity and MASH-related F3 liver fibrosis. The realization of this benefit requires weight loss maintenance longer than one year. Larger prospective studies are needed to determine how weight loss impacts other patient-centered outcomes such as mortality, hepatic decompensation, and hepatocellular carcinoma in patients with obesity and MASH-related F3 liver fibrosis.

## 1. Introduction

Metabolic dysfunction-associated steatotic liver disease (MASLD), formerly non-alcoholic fatty liver disease (NAFLD), is a chronic liver disease that develops in the absence of other causes of secondary hepatic fat accumulation, such as significant alcohol intake and hereditary liver disorders [1,2]. MASLD begins with hepatic steatosis where fat accounts for more than 5% of liver weight but without evidence of hepatocellular injury. Some patients develop the progressive form of the disease, termed metabolic dysfunction-associated steatohepatitis (MASH), previously known as non-alcoholic steatohepatitis, marked by hepatic inflammation and hepatocellular ballooning [2,3]. Ongoing inflammation can lead to liver fibrosis, an important prognostic factor in chronic liver diseases including MASLD. Liver fibrosis is commonly classified into five stages from the absence of fibrosis (F0) to cirrhosis (F4) [4,5]. It is widely acknowledged that a higher degree of fibrosis is associated with worse disease burden from MASH [4,6,7]. In particular, the risk of liver-related complications and all-cause mortality significantly increases once patients progress from F3 to F4 fibrosis (cirrhosis) [7,8]. It is estimated that about 20% of patients with MASH-related F3 fibrosis progress to cirrhosis in 2 years [9].

MASLD is closely associated with cardiometabolic syndrome that encompasses several criteria including elevated body mass index (BMI) (especially central obesity), hypertension, impaired glucose tolerance, and dyslipidemia. Fat deposition in the liver is thought to play a pivotal role in the development of insulin resistance, suggesting that MASLD is not a mere manifestation but could also be a driver of metabolic syndrome [10,11]. The global prevalence of MASLD is estimated to be 25% and has been rising over the past decade, mirroring the obesity pandemic [11]. More specifically, MASLD and MASH affect 30% and 5% of the US population, respectively. MASH is the fastest-growing etiology of liver cirrhosis in the United States, and MASH cirrhosis is on track to become the leading indication for liver transplant in the US [12].

Currently, weight loss and lifestyle modification are the mainstay treatment for MASLD [1]. Multiple studies have demonstrated that weight loss improves liver histology, biochemistry, and metabolic syndrome profiles, such that 3–5% weight loss can improve hepatic steatosis. A greater amount of weight loss (7–10%) is required to achieve improvements in the histopathologic features of MASH, including fibrosis [13,14,15]. However, the majority of patients in these studies had lower fibrosis stages at baseline. For example, 89% of patients enrolled in the trial by Vilar-Gomez et al. (2015) had F0–F2 fibrosis stage at baseline, and the participants in the study by Promrat et al. (2010) had a mean baseline fibrosis stage of 1.5 [14,15]. More research is needed to determine the impact of weight loss on disease progression trajectory in patients with advanced stages of MASH-related fibrosis. This multi-center retrospective study aimed to help address this knowledge gap by examining the effect of weight loss on the preventive development of cirrhosis among patients who have obesity as well as MASH-related F3 liver fibrosis.

## 2. Materials and Methods

### 2.1. Study Design

**Figure 1:**
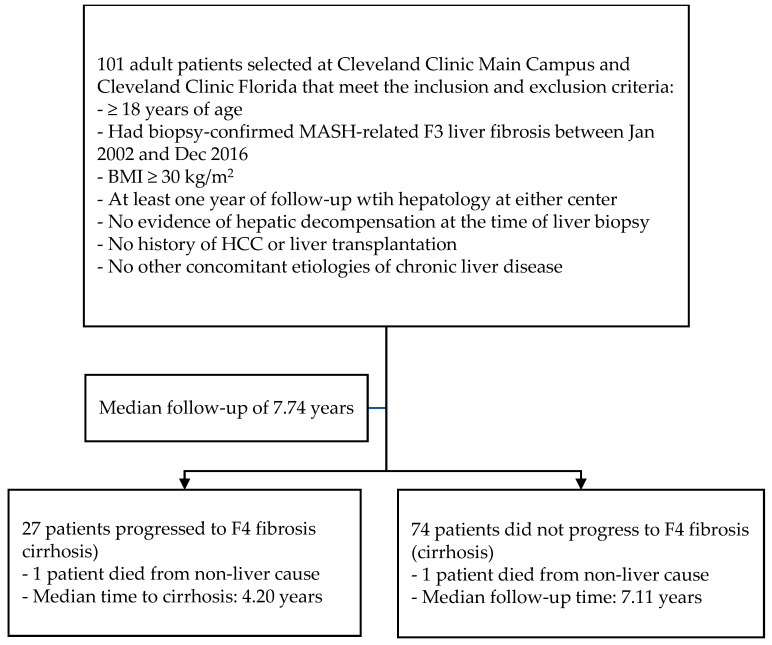
Study Flowchart.

We retrospectively identified all adult patients with obesity (BMI ≥ 30 kg/m^2^) and biopsy-proven MASH with F3 liver fibrosis between January 2002 and December 2016 at two quaternary liver transplant centers—Cleveland Clinic Main Campus (Cleveland, OH, USA) and Cleveland Clinic Florida (Weston, FL, USA) (Figure 1). Patients were excluded if they had evidence of hepatic decompensation, including ascites, hepatic encephalopathy, or variceal bleeding at baseline, which represents the time of the initial liver biopsy. Patients were also excluded if they had a prior history of hepatocellular carcinoma (HCC), had received a liver transplant, or had other concomitant etiologies of chronic liver disease, including significant alcohol consumption (>210 g of alcohol per week for men and >140 g per week for women for 2 years or longer), chronic viral hepatitis, autoimmune liver disease, hereditary liver disease, and hepatotoxic drug use. Patients also needed to have at least one year of follow-up with hepatology at either transplant center after MASH-related F3 diagnosis. A total of 101 patients met all the abovementioned criteria and were included in the study. They were retrospectively followed until 31 December 2020 or the date of their last clinical visit, whichever occurred earlier. The study protocol was approved by the Institutional Review Board (IRB) at the Cleveland Clinic.

### 2.2. Diagnostic Criteria and Data Collection

All liver biopsies were evaluated by liver pathologists to determine the presence of MASH and fibrosis stage using the NAFLD Clinical Research Network (NAFLD-CRN) scoring system [4]. Baseline demographic, laboratory, and pathology data were collected via medical record review. Patients’ baseline model for end-stage liver disease-sodium (MELD-Na) and Child-Turcotte-Pugh (CTP) scores were calculated. The presence of baseline comorbidities (hypertension, type 2 diabetes mellitus, and dyslipidemia) associated with metabolic syndrome and baseline laboratory metabolic profile (low-density lipoprotein [LDL], high-density lipoprotein [HDL], triglyceride [TC], and hemoglobin A1C [HbA1C]) were collected. Self-reported alcohol use and smoking status were collected and recorded as either ‘Never’ or ‘Past or Current’ use. The use of vitamin E and pentoxifylline, which have been shown to improve liver histology in MASH, statin, which is recommended to reduce cardiovascular events among MASH patients, and glucagon-like-peptide 1 receptor agonist (GLP-1 RA), which has been proven to be highly effective in promoting weight loss, were also included [1].

### 2.3. Study Endpoint

The development of cirrhosis during follow-up period was the outcome of interest in this study and was defined as either the presence of cirrhosis on repeat liver biopsy or liver stiffness >12 Kpa on transient elastography (Fibroscan^®^, Echosens, Westborough, MA, USA) plus nodular radiologic appearance of the liver and thrombocytopenia (platelet < 160 K) [16]. Weight and BMI at baseline and 1 year after the initial liver biopsy that conferred the diagnosis of MASH-related F3 fibrosis were collected for all patients. For those who subsequently developed cirrhosis, weight at cirrhosis diagnosis was collected; otherwise, weight at the end of follow-up was used. Relative to baseline weight, percentage of weight loss at 1 year and at event were calculated, where event is defined as the time of cirrhosis diagnosis for those who developed cirrhosis or at the end of follow-up for the remaining patients. Percentage weight loss was further categorized into three levels, namely no weight loss or weight gain, 0–5% weight loss, and >5% weight loss. A positive value for percentage weight loss represents weight loss, whereas a negative value represents weight gain.

### 2.4. Statistical Analysis

Demographic categorical and continuous variables were presented in frequency (%) and median with interquartile range (IQR), respectively. Differences in patient characteristics between those with and without the development of cirrhosis were presented by descriptive analysis. Unadjusted univariable Cox regression models with cause-specific hazard function were used to assess the association between each risk factor and time to event (where event refers to the time of cirrhosis diagnosis in patients who developed cirrhosis or the end of follow-up in patients who did not), with competing event of death.

To assess the association between weight loss groups and cirrhosis development, a multivariable competing risk regression model was performed. Covariates, which are baseline age, BMI, MELD-Na score, and diabetes, were also adjusted in the models, with weight loss at event groups as the time-dependent variables. Due to the limited number of events for some predictors, the model was adjusted by Firth’s penalized method to account for overfitting. 

In multivariable model development, the multivariable imputation by chained equation (MICE) was performed to impute missing values to conduct a complete dataset for variable selection. The stepwise variable selection method based on Akaike information criterion was used to choose the final model. Variables with a large proportion of missing values (>5%), that were unbalanced between weight loss groups, or that were highly correlated with other variables were excluded from the final model. The scaled Schoenfeld residual and the Cox–Snell residual were used to check the Cox proportional hazard survival model’s assumptions and goodness-of-fit. A two-sided α of less than 0.05 was considered statistically significant for this study. Data were managed and analyzed using R software (version 4.3.1, Vienna, Austria).

## 3. Results

Table 1 summarizes the characteristics of the 101 patients included in this study at baseline and throughout the follow-up. The cohort consisted of 72 (71%) women and 29 (29%) men. The median follow-up period for all patients was 92 months. The median baseline age at initial liver biopsy which showed MASH with F3 liver fibrosis was 53.65 years. The majority of patients (64%) had never used alcohol and 36 (36%) reported to have some, but not significant, alcohol use history. Nearly one-half of patients (48%) had never smoked. All patients had a CTP score of 5 at baseline. The median baseline MELD-Na score was 6.0. Baseline cardiometabolic co-morbidities were common: 66 (65%) patients had diabetes; 81 (80%) had hypertension; and 75 (74%) had hyperlipidemia. The metabolic biochemistry panel suggests that these co-morbidities were overall managed fairly well at baseline: median TC was 165 mg/dL, median LDL was 102.50 mg/dL, median HDL was 42.50 mg/dL, and median HbA1C was 6.5. A minority of patients used pharmacological therapies pertinent to MASH: 35 (35%) used a statin, 25 (25%) used pentoxifylline, 30 (30%) used vitamin E, and only 5 (5.0%) used GLP-1 RA.

The median baseline weight and BMI were 98.00 kg and 34.38, respectively. At one year after F3 diagnosis, the median weight decreased to 91.20 kg, representing a median weight loss of 1.96%. A total of 27 (27%) patients developed cirrhosis during follow-up, and these patients had a median weight of 90.2 kg at baseline and 87.80 kg at the time of cirrhosis diagnosis. The remaining 74 patients who did not develop cirrhosis had a median weight of 103.10 kg at baseline and 90.25 kg at the end of follow-up period. Only two (2%) patients died during follow-up, one of whom developed cirrhosis prior to death. Both patients died from non-liver causes.

Univariate analysis was performed to assess the association between multiple risk factors and the progression to cirrhosis, and the results are presented in Table 2. For patients who progressed to cirrhosis, weight at the diagnosis of cirrhosis was used to calculate weight loss at event in the analysis, whereas weight at the end of follow-up was used for the remaining patients who did not develop cirrhosis. Nearly three-quarters of patients (73%) did not progress to cirrhosis. Patients who did not progress to cirrhosis had greater weight loss at the end of follow-up than those who did develop cirrhosis (median 6.23% vs. −0.06%, *p* = 0.027), and a higher proportion of patients who did not progress to cirrhosis achieved >5% weight loss at the end of follow-up (59% vs. 30%, *p* = 0.045). Patients who did progress to cirrhosis had higher median LDL levels than those who did not progress to cirrhosis (123.00 [102.75, 143.75] vs. 101.50 [81.00, 119.25]). The two patient groups did not differ on any other variables in univariate analysis.

Multivariable analysis results (Table 3) also showed that, after adjusting for baseline age, BMI, MELD-Na score, and diabetes, patients with >5% weight loss at the end of follow-up had a lower hazard of progressing to cirrhosis compared to patients with no weight loss or weight gain (HR: 0.29, 95%, CI: 0.08–0.96). Similarly, patients with type 2 diabetes (HR: 3.24, 95%, CI: 1.21–8.67) and higher LDL levels (HR: 1.02, 95%, CI: 1.01–1.04) had higher hazards of progressing to cirrhosis.

## 4. Discussion

Multiple studies have demonstrated that weight loss can improve hepatic steatosis and histological features of MASH in a positive dose-response fashion [14,15]. However, since previous studies lacked a sizable percentage of patients with F3 liver fibrosis, little is known about the impact of weight loss on these patients. These studies focused on liver histology as the primary outcome and have a relatively short study duration ranging from 3 to 12 months. There is a paucity of data on patient-centered outcomes, such as the prevention of cirrhosis development in MASLD. As such, our multi-center study examined the effect of weight loss on the prevention of progression to cirrhosis in a sample exclusively composed of patients with obesity and MASH-related stage F3 liver fibrosis over a long follow-up period (median 7.74 years) to better capture the course of this chronic disease.

The adjusted multivariate model showed that weight loss of >5% at follow-up was significantly associated with a decreased risk of developing cirrhosis. Patients who developed cirrhosis had a median weight gain of 0.06% at time of cirrhosis diagnosis, while those who did not develop cirrhosis had a median weight loss of 6.23%. This observation is in line with the results of the prospective study by Vilar-Gomez et al. (2015) that weight loss of at least 7% is required to achieve steatohepatitis resolution and >2 point improvement in NAS score [14]. Not only could weight loss lead to histologic improvement in MASH, as demonstrated in previous studies, but findings from our study suggest that >5% weight loss could help prevent or delay the progression from F3 fibrosis to cirrhosis. Of note, patients who did not develop cirrhosis had a much longer median time-to-event period (7.11 years) than those who did (4.20 years), further supporting the protective effect of weight loss.

In the univariate analysis, weight loss at 1 year after F3 diagnosis was not associated with a lower risk of progression to cirrhosis, even among patients who achieved >5% weight loss. It is plausible that longer than one year is needed for weight loss to halt or regress fibrosis. Another explanation is that some patients may not have sustained weight loss after the first year, and the initial benefit from weight loss in preventing or delaying progression to cirrhosis eventually dissipated. Long-term maintenance of weight loss is a challenging task and often requires longitudinal care involving dietary and physical activity coaching, as well as cognitive behavioral interventions [17,18]. Our findings could be used to facilitate clinical management and counseling when caring for patients with obesity and MASH-related F3 liver fibrosis. Clinicians could inform patients that the benefits of weight loss in delaying or preventing the progression to cirrhosis are unlikely to occur immediately, encourage patients to engage in weight loss regimens beyond one year, and emphasize the importance of long-term, sustained weight loss. Altogether, this may further boost patients’ efforts to continue losing weight.

There are multiple options that clinicians can choose from to manage patients with obesity and MASH-related F3 liver fibrosis. Bariatric surgery has been shown to achieve and maintain >10% of weight loss as well as reduce liver-related complications and cardiovascular events in patients with MASH and obesity [19]. Several medications to treat obesity are FDA approved. For example, multiple studies have shown that GLP-1 RA could promote a greater amount of weight loss than lifestyle modification alone [20,21]. These agents could become promising treatments for MASH and associated metabolic dysfunctions, especially when used in combination with lifestyle modifications. There are also several phase III trials on new drugs to treat diabetes and obesity, as well as MASLD, MASH, and fibrosis regression in the liver.

The multivariate analysis also showed that type 2 diabetes is significantly associated with an increased hazard of progression to cirrhosis in patients with MASH-related F3 fibrosis. This is unsurprising, given the strong evidence of a bidirectional relationship between type 2 diabetes and MASLD and an increased risk of both diseases in patients with obesity. Type 2 diabetes and the associated chronic inflammation and insulin resistance could hasten the disease progression of MASLD to its more severe forms, such as MASH and MASH-related cirrhosis [1,22]. Through the same underlying metabolic derangement, dyslipidemia, including the overproduction of triglyceride and LDL, as well as lower levels of HDL, is also closely linked to MASLD [22]. This likely explains the similar association between elevated LDL and higher risk of progression to cirrhosis observed in this study. However, the clinical significance of this finding is uncertain given the low hazard ratio of 1.02 and the fact that aggressive cardiovascular risk factor management is already a routine practice in the care of MASLD patients [1]. The lack of significant association between cirrhosis development and other lipid parameters, such as HDL and TC, is likely attributed to the relatively small cohort size and the fact that all patients shared many common features in their metabolic profiles.

The study had several limitations and strengths. While epidemiology studies have shown that, globally, MASH-related advanced liver fibrosis is more prevalent in women, with a male/female ratio of 1:1.45, this patient cohort has a more pronounced gender imbalance towards women, consisting of 29 males and 72 females [23]. This likely reflects the gender distribution of patients with MASH-related advanced liver fibrosis at the two medical centers where data collection took place. There is a lack of epidemiology data on the local prevalence of MASH or MASH-related fibrosis in the states of Ohio and Florida to account for this gender bias, which can affect the applicability of the results in regions with different patterns of gender distribution. Another limitation is that we were unable to collect information on patients’ weight loss regimens, including lifestyle modification and pharmacologic treatment, from chart review. While there is some evidence of heritability in MASLD, family histories of chronic liver disease and metabolic syndrome were unavailable for data extraction [24]. There was also a large variation in the length of follow-up, given that the time of the index liver biopsy spanned a wide range from January 2002 to December 2016. As such, some patients could have been incorrectly deemed to be free of cirrhosis if not given enough time to allow natural disease progression to occur. The longer median time-to-event time in patients who did not develop cirrhosis than those who did reduces but does not eliminate this risk. Lastly, retrospective studies and small sample sizes are inherently prone to biases and an inability to account for all confounding factors. Nonetheless, several confounding variables were adjusted for in the modeling, and adjustments were also made to account for overfitting. The study was multi-centered and had an extended observation period. Our findings are consistent with the conclusions of previous clinical trials that studied the effect of weight loss on the histopathology of MASLD and further support the recommendations of multiple professional societies that weight loss plays a central role in the management of MASLD and MASH [1,14,25].

## 5. Conclusions

In conclusion, this study showed that weight loss >5% has the potential to prevent disease progression to cirrhosis in patients with obesity and MASH-related F3 liver fibrosis, but weight loss sustained for longer than one year is likely needed to realize this benefit. It is advisable for patients to be counseled on the importance of and strategies to achieve long-term maintenance of weight loss. Type 2 diabetes is also associated with a higher hazard of progression to cirrhosis in patients with obesity and MASH-related F3 fibrosis. Moving forward, prospective studies with larger sample sizes are needed to estimate the duration of sustained weight loss required to halt the progression of, or even reverse, F3 liver fibrosis and to more accurately determine the effect of weight loss on other patient-centered outcomes, such as mortality, hepatic decompensation, and HCC, among patients with obesity and MASH-related F3 liver fibrosis.

## Figures and Tables

**Table 1:** Baseline characteristics and progression to cirrhosis.

Characteristic	*n* = 101 ^1^
Gender Female Male	72 (71%) 29 (29%)
Body mass index (kg/m^2^)	34.38 (30.88, 40.40)
Age at diagnosis (year)	53.65 (46.52, 62.07)
Alcohol status Never Past or Current	65 (64%) 36 (36%)
Smoking status Never Past or Current	48 (48%) 53 (52%)
MELD-Na score at baseline	6.0 (6.00, 6.00)
Child-Turcotte-Pugh score at baseline	5.0 (5.00, 5.00)
Hypertension	81 (80%)
Hyperlipidemia	75 (74%)
Diabetes mellitus	66 (65%)
Metabolic syndrome	85 (84%)
Baseline triglyceride (mg/dL)	165.00 (115.00, 216.50)
Baseline LDL (mg/dL)	102.50 (82.00, 130.75)
Baseline HDL (mg/dL)	42.50 (35.00, 50.00)
Baseline HbA1c (%)	6.50 (6.00, 7.50)
Previous bariatric surgery	20 (20%)
Statin user	35 (35%)
Pentoxifylline user	25 (25%)
Vitamin E user	30 (30%)
GLP-1 RA user	5 (5.0%)
Any pharmacologic ^a^	66 (65%)
Baseline weight (kg)	98.00 (84.80, 115.00)
Weight at 1 year after F3 diagnosis (kg)	91.20 (81.50, 111.40)
Weight loss percentage at 1 year after F3 diagnosis (%)	1.96 (−0.64, 8.53)
Weight loss levels at 1 year after F3 diagnosis 0 or weight gain 0–5% >5%	36 (36%) 33 (33%) 32 (32%)
Weight at the last follow-up visit (kg)	89.80 (77.20, 107.20)
Weight at the last follow-up visit (kg)	89.80 (77.20, 107.20)
Weight loss at the last follow-up visit (%)	6.07 (−0.65, 12.69)
Had pre-existing cardiovascular disease at baseline	10 (9.9%)
Had cardiovascular event during follow-up	16 (16%)
Weight at the cardio event (kg) ^b^	97.05 (82.67, 105.60)
Progression to cirrhosis	27 (27%)
Weight at cirrhosis diagnosis (kg) ^c^	87.80 (81.15, 101.45)
All-cause mortality	2 (2.0%)
Follow-up Time (year)	7.74 (5.17, 9.85)

^1^ *n* (%); Median (OQR). ^a^ Any pharmacologic of: statin, pentoxifylline, vitamin E, or GLP-1RA. ^b^ Only for patients who had cardiovascular event during follow-up. ^c^ Only for patients who developed cirrhosis during follow-up.

**Table 2:** Univariable analysis and characteristics comparisons on the outcome of progression to cirrhosis.

	Progression to Cirrhosis Status		Univariable Analysis Results		
Characteristic, *n* = 101	No, *n* = 74 ^1^	Yes, *n* = 27 ^1^	HR ^2^	95% CI ^2^	*p*-value
Gender Female Male	53 (72%) 21 (28%)	19 (70%) 8 (30%)	__ 0.98	__ 0.42, 2.25	0.96
Body mass index (kg/m^2^)	35.63 (31.03, 40.84)	33.98 (28.51, 36.98)	0.98	0.93, 1.04	0.50
Age (year)	53.44 (46.26, 59.95)	55.71 (48.35, 64.33)	1.03	0.98, 1.07	0.22
Alcohol status Never Past or Current	46 (62%) 28 (38%)	19 (70%) 8 (30%)	__ 0.68	__ 0.29, 1.57	0.35
Smoking status Never Past or Current	33 (45%) 41 (55)	15 (56%) 12 (44%)	__ 0.57	__ 0.26, 1.24	0.15
MELD-Na Score	6.00 (6.00, 6.00)	6.00 (6.00, 6.00)	1.21	0.72, 2.03	0.50
Hypertension No Yes	13 (18%) 61 (82%)	7 (26%) 20 (74%)	__ 0.96	__ 0.38, 2.44	0.93
Type 2 diabetes mellitus No Yes	28 (38%) 46 (62%)	7 (26%) 20 (74%)	__ 1.82	__ 0.76, 4.34	0.16
Metabolic syndrome No Yes	12 (16%) 62 (84%)	4 (15%) 23 (85%)	__ 1.34	__ 0.46, 3.95	0.58
Baseline TC (mg/dL)	162.50 (125.00, 219.50)	171.00 (110.25, 204.00)	1.00	1.00, 1.00	0.94
Baseline LDL (mg/dL)	101.50 (81.00, 119.25)	123.00 (102.75, 143.75)	1.02	1.00, 1.03	0.01
Baseline HDL (mg/dL)	42.00 (34.75, 49.25)	44.00 (38.00, 51.50)	1.00	0.96, 1.04	0.96
Baseline HbA1c (%)	6.45 (6.00, 7.48)	6.60 (6.00, 7.60)	1.18	0.84, 1.66	0.34
Previous bariatric surgery No Yes	56 (76%) 18 (24%)	25 (93%) 2 (7.4%)	__ 0.35	__ 0.08, 1.47	0.094
Statin user No Yes	48 (65%) 26 (35%)	18 (67%) 9 (33%)	__ 1.13	__ 0.50, 2.56	0.77
Pentoxifylline user No Yes	55 (74%) 19 (26%)	21 (78%) 6 (22%)	__ 0.89	0.39, 2.46	0.97
Vitamin E user No Yes	53 (72%) 21 (28%)	18 (67%) 9 (33%)	__ 1.21	0.52, 2.79	0.66
GLP-1 RA user No Yes	69 (93%) 5 (6.8%)	27 (100%) 0 (0%)	__ 0.00	__ 0.00, Inf	0.25
Any pharmacologic ^a^ No Yes	27 (36%) 47 (64%)	8 (30%) 19 (70%)	__ 1.33	0.58, 3.07	0.49
Baseline weight (kg)	103.10 (88.03, 117.15)	90.20 (82.60, 102.30)	1.0	0.98, 1.03	0.50
Weight loss at 1 year after F3 diagnosis (%)	2.53 (−0.28, 10.27)	0.41 (−1.55, 4.67)	0.98	0.93, 1.03	0.32
Weight loss levels at 1 year after F3 diagnosis 0% or weight gain 0–5% >5%	23 (31%) 25 (34%) 26 (35%)	13 (48%) 8 (30%) 6 (22%)	__ 0.74 0.63	__ 0.30, 1.81 0.24, 1.68	0.61
Weight loss percentage at event ^b^ (%)	6.23 (−0.13, 14.07)	−0.06 (−3.31, 7.24)	0.96	0.92, 1.00	0.027
Weight loss levels at event 0% or weight gain 0–5% >5%	19 (26%) 11 (15%) 44 (59%)	14 (52%) 5 (19%) 8 (30%)	__ 0.97 0.35	__ 0.35, 2.72 0.14, 0.87	0.045
Time to event (years)	7.11 (4.93, 9.67)	4.20 (2.83, 8.07)			

^1^ *n* (%); Median (IQR). ^2^ HR = hazard ratio, CI = confidence interval. ^a^ Any pharmacologic of statin, pentoxifylline, vitamin E, or GLP-1 RA. ^b^ Event refers to progression to cirrhosis for the 27 patients who progressed to cirrhosis during follow-up or end of follow-up for the 74 patients who did not progress to cirrhosis.

**Table 3:** Multivariable Cox proportional hazard model on the outcome of progression to cirrhosis.

Characteristics	HR ^1^	95% CI ^2^	*p*-Value
Baseline age (years)	1.01	0.95, 1.06	0.82
BMW at baseline (kg/m^2^)	1.01	0.94, 1.09	0.77
MELD-Na score	1.36	0.85, 2.18	0.10
Type 2 diabetes mellitus: Yes vs. No	3.24	1.21, 8.67	0.022
LDL (mg/dL)	1.02	1.01, 1.04	0.008
Weight loss levels at event ^a^ 0–5% vs. No weight loss or weight gain >5% vs. No weight loss or weight gain	0.94 0.29	0.31, 2.86 0.08–0.96	0.91 0.043

^1^ HR = Hazard Ratio. ^2^ CI = confidence interval. ^a^ Event refers to progression to cirrhosis for the 27 patients who progressed to cirrhosis during follow-up or end of follow-up for the 74 patients who did not progress to cirrhosis. The model was adjusted by Firth’s penalized method.

## Data Availability

The patient data presented in this article are not readily available because of ethical IRB-related privacy reasons. Consideration for access to data can be submitted to the corresponding author.
